# The Maize Class-I SUMO Conjugating Enzyme ZmSCE1d Is Involved in Drought Stress Response

**DOI:** 10.3390/ijms21010029

**Published:** 2019-12-19

**Authors:** Huanyan Wang, Meiping Wang, Zongliang Xia

**Affiliations:** 1 College of Life Science, Henan Agricultural University, Zhengzhou 450002, China; 2 Library, Henan Agricultural University, Zhengzhou 450002, China; 3 Collaborative Innovation Center of Henan Grain Crops and Key Laboratory of Wheat and Maize Crop Science, Henan Agricultural University, Zhengzhou 450002, China

**Keywords:** maize, drought, antioxidant enzyme, sumoylation

## Abstract

Post-translational modification of cellular proteins by sumoylation plays a vital role in stress responses of plants. However, the mechanisms underlying the sumoylation’s involvement in stress responses in crop species remain largely unknown. Herein, a maize class-I SUMO conjugating enzyme gene (*ZmSCE1d*) was identified, whose expression was upregulated upon drought stress. Over-expression of *ZmSCE1d* in transgenic *Arabidopsis* plants increased SUMO conjugates and improved drought tolerance. The *ZmSCE1d*-transgenic plants showed higher antioxidant enzyme activities, but lower reactive oxygen species and lipid peroxidation upon drought stress. Furthermore, transcripts of several drought-responsive genes were significantly elevated, as revealed by qPCR in the transgenic lines. Taken together, these data have demonstrated that *ZmSCE1d* overexpression improved drought tolerance likely by regulating sumoylation levels, antioxidant capability, and drought-responsive gene expression in transgenic plants. This study may facilitate our understanding of the mechanisms underlying SCE-mediated sumoylation under drought stress and accelerate genetic improvement of crop plants tolerant to drought stress by manipulating the SUMO system.

## 1. Introduction

Drought is a key environmental stress factor that significantly affects crop growth, development, and productivity [1,2]. To acclimate and tolerate the adverse environment, plants have to make significant changes in post-translational modification (PTM) of proteins [3]. Sumoylation by the small ubiquitin-related modifier (SUMO) protein family is an essential PTM regulatory process and is involved in developmental and stress responses [4,5,6,7].

Sumoylation occurs through a three-step reaction cascade in which SUMO is covalently attached to target proteins by three enzymes E1, E2, and E3 similar to ubiquitylation [8,9]. The E1 activating enzyme (SAE) promotes activation of SUMO in an ATP-dependent manner and the E2 conjugating enzyme (SCE) mediates conjugation of adenylated SUMO to target proteins. The SCE specifically recognizes substrates in the absence of a SUMO E3 ligase [10]. Thereby, the E3 ligases are not essential for the conjugation process [11,12]. However, SUMO E3 ligases have an important role in substrate specificity by transferring SUMO from the SCE to target proteins [12].

In higher plants, sumoylation plays a central role in developmental, hormonal, and environmental stress responses [13,14]. Up to now, eight SUMO members, two SUMO E1 enzymes SAEs, one E2 enzyme SCE, and two SUMO E3 ligases have been identified in *Arabidopsis* genome [15,16]. At present, most studies have been focused on the functional characterization of sumoylation components in *Arabidopsis*. As examples, AtSUMO1/2 over-expression enhanced sumoylation status, reduced ABA sensitivity, and up-regulated ABA-responsive gene transcripts in *Arabidopsis* [17]. Interestingly, it was reported that *Arabidopsis* SUMO E3 ligase AtSIZ1 had a broader and pleiotropic role in plants. The AtSIZ1 not only controls cell growth and plant development, but also regulates abscisic acid signaling, phosphate deficiency, salicylic acid-mediated innate immunity, and abiotic stresses such as heat, freezing, salt, drought, and excess copper stress responses by affecting sumoylation of specific target proteins [4,18,19,20,21,22,23,24,25,26,27,28]. In addition, mutation of the AtSIZ1 disrupts mature female gametophyte and knockout of SUMO conjugating enzyme AtSCE1 or AtSUMO1/2 causes embryo lethality, implying that these sumoylation machinery components are necessary for plant growth and development [24,29,30].

In crop plants, functional studies of sumoylation components are still in its infancy [5,31,32]. To our knowledge, most of studies were centered on the SUMO E3 ligases in crops. For instances, the rice SUMO E3 ligase OsSIZ1 regulates phosphate and nitrogen starvation responses, spikelet development and fertility [32,33,34]. In soybean, two SUMO E3 ligase GmSIZ1a and GmSIZ1b mediate sumoylation and positively regulate vegetative growth [35]. These findings indicate that the E3 ligase SIZ1 also plays crucial roles in developmental and stress responses in crop plants.

Although the SCEs have the ability of enhancing substrate specificity, the role of SCEs in plants is poorly understood. The SCE is encoded by a single gene in *Arabidopsis*, whereas at least two SCE genes were found in other plant species [18,36]. Rice has three SCE genes (OsSCE1a, OsSCE1b, and OsSCE1c) with slightly different sequences, which may be considered a monocot-specific subgroup [36]. Moreover, OsSCE1a is found to be involved in carbohydrate metabolism and OsSCE1c participated in drought tolerance in rice [37], indicating that OsSCEs may be involved in different cellular processes.

Maize (*Zea mays* L.) is an important staple and feed crop that is adversely affected in growth and yield by environmental stresses. Unfortunately, molecular characterization of the maize sumoylation machinery-related genes in response to abiotic stresses is few. In maize, seven *SCE1* genes (*SCE1a-SCE1g*) have been identified that were clustered into two distinct subfamilies (class-I and class-II) [38]. The maize class I members include SCE1a to SCE1d and the class II clade includes SCE1e to SCE1g [38]. Most recently, a putative SUMO conjugating enzyme ortholog from maize class-II (ZmSCE1e) in our laboratory has been reported to be involved in salt and drought tolerance [39]. To comprehensively understand the function of the ZmSCEs, we analyzed the expression patterns of all the members of the class-I SCEs in maize under water deficit, and found that ZmSCE1d might be involved in drought response and tolerance using the transgenic approach.

## 2. Results

### 2.1. Molecular Characterization of the Maize Class-I SUMO Conjugating Enzyme Genes

In maize, seven *SCE1* genes (named *SCE1a*, *b*, *c*, *d*, *e*, *f*, and *g*) were clustered into two distinct subfamilies (class-I and class-II). The maize class-I members include SCE1a to SCE1d and the class-II clade includes SCE1e to SCE1g [38,39]. The ORF of each class-I member consists of 483 nucleotide acids and encodes a protein of 160 amino acids. Moreover, these class-I ZmSCE1s have higher identity (>90%) each other, as revealed by amino acid sequence alignments.

**Figure 1:**
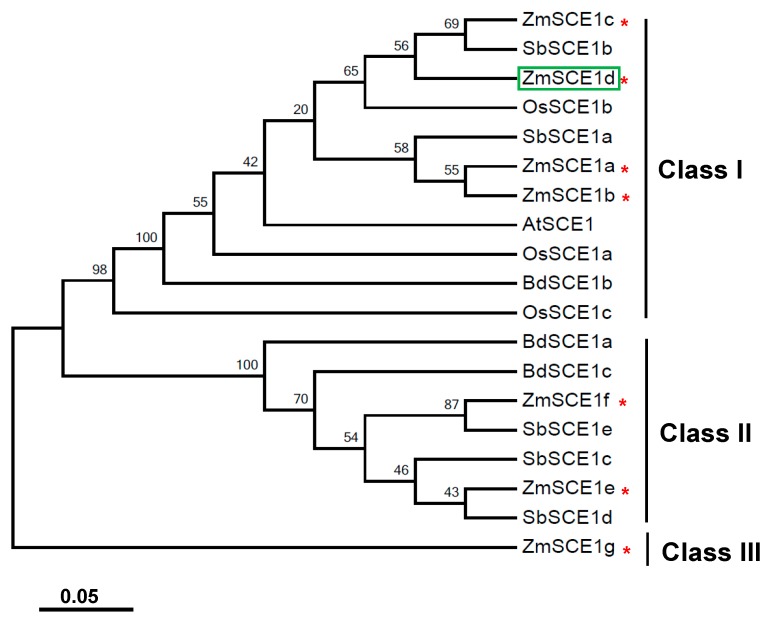
Phylogenetic analysis of SCE1 proteins from *Zea mays* and other cereal species. Phylogenetic tree based on E2 conjugating enzyme (SCE) 1 protein sequences from rice, *Sorghum*, wild wheat, maize and *Arabidopsis*. *Zea mays* (ZmSCE1a-ZmSCE1g), *Sorghum bicolor* (SbSCE1a-SbSCE1e), *Oryza sativa* (OsSCE1a-OsSCE1c), *Brachypodium distachyon* (BdSCE1a-BdSCE1c), and *Arabidopsis thaliana* (AtSCE1). The numbers at the nodes indicate bootstrap values, which were calculated based on 500 replications. The red * indicates the maize SCE1a to SCE1g. The tree was constructed using the neighbor-joining method. The SCE1 protein sequences from 5 species were downloaded from the report by Augustine et al. (2016) [38].

A phylogenetic tree was established based on SCE1 proteins from maize and other cereal species, such as rice, sorghum, and wild wheat (Figure 1). As shown in Figure 1, these cereal SCE1s were clustered into three distinct subfamilies (class-I, class-II, and class-III). Class-I members include four maize isotypes (ZmSCE1a to ZmSCE1d), three rice isotypes (OsSCE1a to OsSCE1c), two Sorghum isotypes (SbSCE1a and SbSCE1b), and one brachypodium isotype (BdSCE1b), besides the *Arabidopsis* AtSCE1.The class-II clade includes two maize isotypes (SCE1e and SCE1f), three sorghum isotypes (SbSCE1c to SbSCE1e), and two brachypodium isotypes (BdSCE1a and BdSCE1c). In contrast, ZmSCE1g on the distinct branch can be classified as the class III. These results indicate that the maize class-I isotypes are similar to the known *Arabidopsis* SCE1, but the class-II and class-III members are cereal-specific isoforms.

### 2.2. Transcript Profiles of the Class-I ZmSCE1 Members during PEG-induced Osmotic Stress in Maize Plants

**Figure 2:**
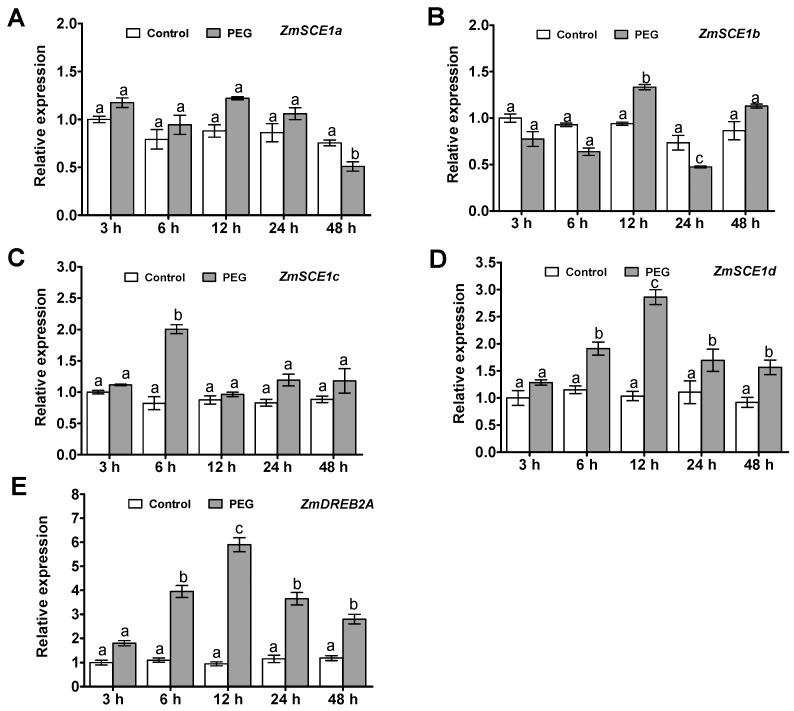
Transcript profiles of class-I *ZmSCE1s* in maize plants under PEG-induced water stress. Changes in transcript levels of *ZmSCE1a* (**A**), *ZmSCE1b* (**B**), *ZmSCE1c* (**C**), *ZmSCE1d* (**D**), and *ZmDREB2A* (**E**) at various time points in response to drought stress in maize plants. Two-week-old maize seedlings were exposed to 0% and 20% PEG6000 for indicated time points (3, 6, 12, 24, and 48 h), and leaf samples were used for qPCR analysis. For each qPCR, the transcript levels of maize reference gene *Ubiquitin* were also evaluated in various samples. For each experiment, three technical replicates were conducted. Data shown are Mean ± SE of three independent experiments. Statistical analysis was performed using ANOVA test (*p* < 0.05) and significant differences are indicated by different letters.

Time-course analysis of the maize class-I *SCE1* genes (*ZmSCE1a*, *b*, *c*, and *d*) transcript levels in maize plants under PEG-induced osmotic stress was conducted by qPCR (Figure 2). Upon PEG treatment, transcripts of the three *SCE1* genes *ZmSCE1a*, *b*, and *c*, had no significant changes during 48 h of the stress, except for at a certain time point (Figure 2A–C). In contrast, the *ZmSCE1d* expression increased significantly at 6 h, and reached a peak at 12 h (~2-fold increase), and then gradually decreased, and still maintained higher levels during 48 h of the stress (Figure 2D).

Meanwhile, to show the effectiveness of the treatment, we examined the expression of the drought- or osmotic-responsive gene *ZmDREB2A* [40] in maize seedlings under PEG6000 treatment by qPCR. As expected, compared to their corresponding controls, significantly increased expression of the stress-inducible marker gene *ZmDREB2A* was observed under PEG-induced osmotic stress (Figure 2E). These data suggest that the class-I *ZmSCE1* genes showed differential response patterns under drought stress. In particular, expression of the *ZmSCE1d* was upregulated upon osmotic stress.

### 2.3. Responses of ZmSCE1d-Transgenic Arabidopsis Plants to Osmotic Stress

To explore physiological function of the *ZmSCE1d*, the binary expression construct pART27 harboring *ZmSCE1d* driven by the CaMV 35S promoter was developed (Figure S1A) and transformed into *Arabidopsis* plants. To this end, six independent homozygous transgenic *Arabidopsis* lines (named L1, L4, L12, L16, L20, and L23) were developed, in which transcript levels of the *ZmSCE1d* were analyzed by qPCR (Figure S1B). Of them, four independent lines (L1, L4, L20, and L23) with higher *ZmSCE1d* transcripts were selected for further functional analysis.

**Figure 3:**
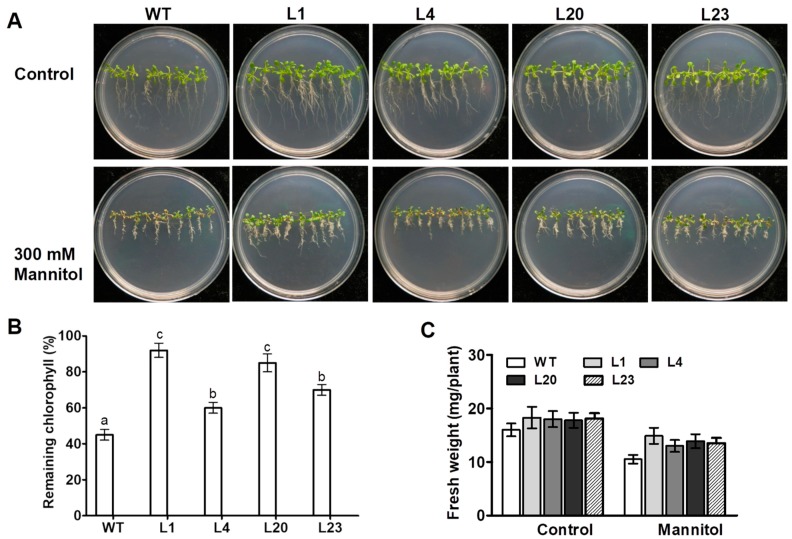
Phenotypes of *ZmSCE1d*-overexpressing and wild type *Arabidopsis* plants under osmotic stress. (**A**) Growth phenotypes of wild-type (WT) and *ZmSCE1*-overexpressing (OE) *Arabidopsis* plants under osmotic stress. Ten-day-old WT and OE (L1, L4, L20, and L23) seedlings vertically growing on 1/2 MS medium supplemented with 0, and 300 mM mannitol for 10 d. (**B**) Relative remaining chlorophyll (%) of 10 day-mannitol-stressed WT and OE plants. Values are mean ± SE, *n* = 20. (**C**) Fresh weight of 10 day-mannitol-stressed WT and OE plants. Values are mean ± SE, *n* = 20. In both B and C, different letters indicate significant differences between WT and OE lines (ANOVA; *p* < 0.05).

To investigate effect of *ZmSCE1d* overexpression on osmotic stress tolerance in plants, ten-day-old WT and four transgenic *Arabidopsis* seedlings were treated separately with 0 or 300 mM mannitol. After 10 days, the WT seedlings showed more chlorosis than the four transgenic lines upon 300 mM mannitol (Figure 3A). In contrast, these transgenic plants seemed to show much better growth vigor than the WT under control conditions (Figure 3A). Chlorophyll content determination showed that the transgenic lines had much higher residual chlorophyll than the WT after 10 days of osmotic stress (Figure 3B). Compared with unstressed plants, fresh weight decreased by 35% in the WT, whereas only reduced by 20% averagely in the transgenic lines upon osmotic stress (Figure 3C). These results evidenced that overexpression of *ZmSCE1d* significantly enhanced osmotic stress tolerance of plants in transgenic *Arabidopsis*.

### 2.4. Responses of ZmSCE1d-Transgenic Arabidopsis Plants to Drought Stress

**Figure 4:**
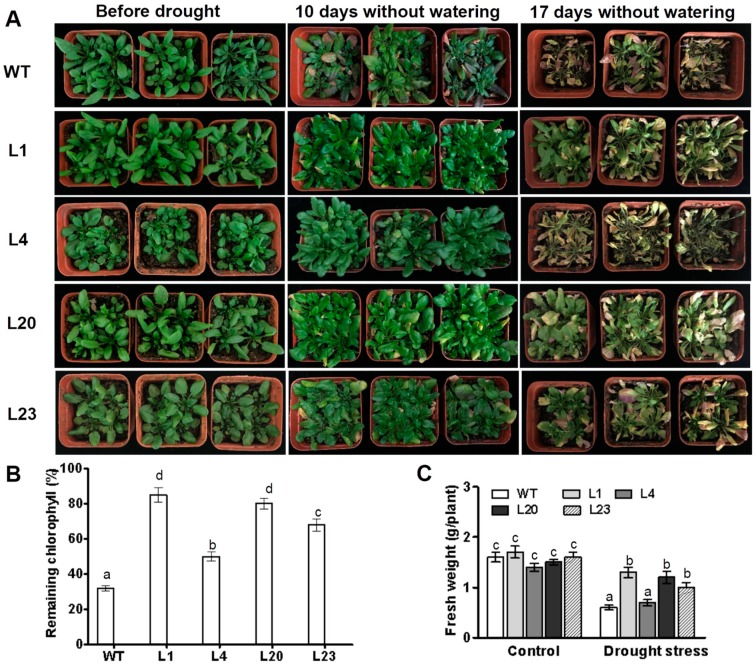
Phenotypes of wild type and *ZmSCE1d*-overexpressing *Arabidopsis* plants under drought stress. (**A**) Drought tolerance of potted wild-type (WT) and overexpressing (OE) *Arabidopsis* plants. Three-week-old WT and transgenic OE (L1, L4, L20, and L23) plants were grown in soil in pots for 17 d without watering. (**B**) Relative remaining chlorophyll (%) of 17 d-drought-stressed WT and OE plants. Values are mean ± SE, *n* = 15. (**C**) Fresh weight of 17 day-drought-stressed WT and OE plants. Values are mean ± SE, *n* = 15. In both (**B**) and (**C**), different letters indicate significant differences between WT and OE lines (ANOVA; *p* < 0.05).

The phenotypes of *ZmSCE1d*-transgenic plants under drought stress in soil were examined. After 10 days without watering, leaves of most WT plants turned yellow and dark purple, whereas the *ZmSCE1d*-transgenic leaves just began to be slightly yellowed (Figure 4A, middle lanes). After 17 days without watering, all WT plants displayed severe wilting (most of the leaves were severely curled and turned yellow), even suffered to be dying, whereas *ZmSCE1d* transgenic lines showed signs of moderate water deficit (at least 50% of upper leaves were still green and fully expanded) (Figure 4A, right lanes). Accordingly, residual chlorophyll in *ZmSCE1d* transgenic lines was significantly higher than that in WT plants (Figure 4B). Compared with their corresponding controls, most of these transgenic lines had less reduction in fresh weight than the WT after drought stress (Figure 4C). These results demonstrated that *ZmSCE1d* overexpression in transgenic *Arabidopsis* also improved drought tolerance.

### 2.5. Changes in Lipid Peroxidation, Reactive Oxygen Species, and Antioxidant Enzyme Activities in ZmSCE1d-Trangenic Plants under Drought Stress

**Figure 5:**
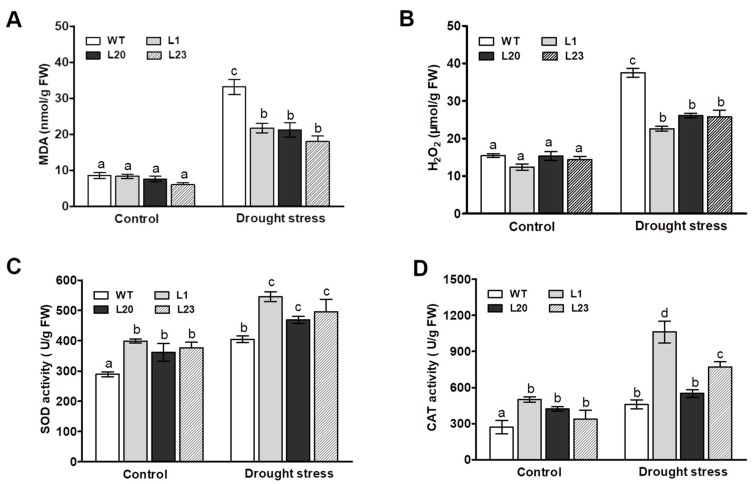
Changes of MDA, H_2_O_2_, and antioxidant enzymes in *ZmSCE1d*-overexpressing and wild type *Arabidopsis* plants under PEG-induced water stress. Four weeks old WT and OE plants (L1, L20, and L23) were treated with distilled water (control) or 20% PEG-6000 solutions, respectively, for 12 h, and then leaf samples were sampled to determine MDA content (**A**), H_2_O_2_ content (**B**), SOD (**C**), and CAT (**D**). Data are means ± SE calculated from three replicates. Bar indicates SE. In these figures, different letters indicate significant differences between WT and OE lines (ANOVA; *p* < 0.05).

We next examined effects of ZmSCE1d overexpression on lipid peroxidation and reactive oxygen species (ROS). MDA content was measured among the WT and three transgenic lines after 12 h of 20% PEG-induced water stress. As shown in Figure 5A, the MDA content in both WT and three transgenic lines was significantly elevated upon drought stress, compared to their corresponding controls. However, the WT plants increased by 280% in MDA content upon PEG treatment, whereas these three transgenic lines only increased by 175% on average (Figure 5A), suggesting that these ZmSCE1d-overexpressing *Arabidopsis* plants suffered less membrane damage than the WT upon drought stress.

H_2_O_2_ levels were then measured in both WT and the three transgenic lines upon drought stress. As shown in Figure 5B, after 12 h of 20% PEG treatment, the H_2_O_2_ levels in both transgenic lines and WT plants markedly increased compared to their corresponding controls (Figure 5B). However, there were differential magnitudes of increases in these types of plants. For example, H_2_O_2_ levels in these three transgenic lines (~75% increase on average) were significantly lower than the WT (~150% increase), showing that WT plants accumulated higher levels of H_2_O_2_ relative to these transgenic lines upon drought stress (Figure 5B). These physiological indices implied that the different levels of lipid peroxidation and ROS accumulation in both WT and transgenic lines could be in correlation with their differential tolerance to drought stress.

We finally examined the activities of two major antioxidant enzymes SOD and CAT between WT and these transgenic lines under drought stress. As shown in Figure 5C, upon 12 h of PEG treatment, compared to their corresponding controls, both WT and these transgenic lines showed significant increases in SOD and CAT activities. Moreover, these transgenic lines showed more magnitudes of increases than the WT plants (Figure 5C,D). Noticeably, these three transgenic lines had relatively higher antioxidant enzyme activities than the WT plants under control conditions (Figure 5C,D). This result, along with changes of MDA and H_2_O_2_ levels, suggested that *ZmSCE1d* overexpression reduced lipid peroxidation and ROS accumulation likely by enhancing activities of major antioxidant enzymes under drought stress.

### 2.6. Changes of Sumoylation Levels in ZmSCE1d-Trangenic Plants under Drought Stress

**Figure 6:**
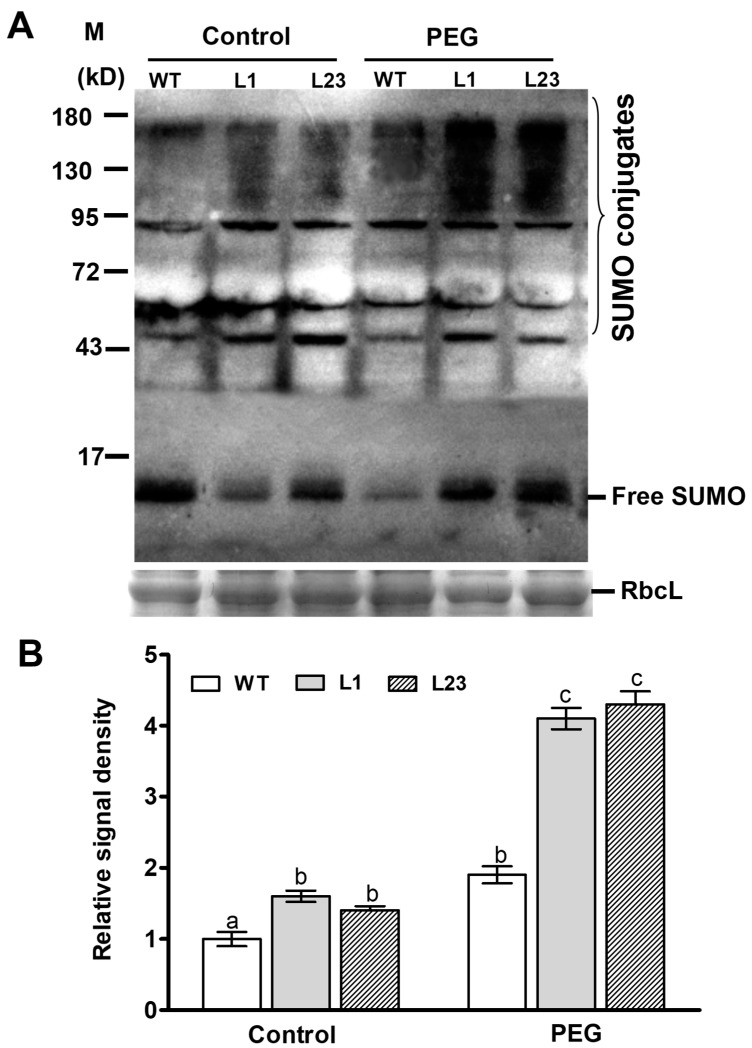
Changes of endogenous sumoylation by the small ubiquitin-related modifier (SUMO) conjugates in *ZmSCE1d*-overexpressing and wild type *Arabidopsis* plants under water stress. (**A**) Levels of SUMO conjugates in *ZmSCE1d*-overexpressing and wild type *Arabidopsis* plants under control or drought stress. Four-week-old potted-WT and OE transgenic lines (L1 and L23) were treated with distilled water (control) or 20% PEG-6000 solutions, respectively, for 6 h, and then leaves were sampled for detection of SUMO conjugates by Western blot using the anti-AtSUMO1 monoclonal antibody. Coomassie blue-stained Rubisco large subunit (RbcL, 55 kD) was used as a loading control. Each lane contained 20 µg of protein. (**B**) Relative signal density of SUMO conjugates in *ZmSCE1d*-overexpressing and wild type plants under PEG-induced water stress. The signal intensity was determined from the blotted membrane between 130 kDa and 180 kDa by the Adobe Photoshop. Relative signal intensity values were shown from the ratios of sumo conjugates intensity to the loading control intensity. Means and SE were from three measurements, and different letters on the histograms indicate significant differences (ANOVA; *p* < 0.05).

To study effect of *ZmSCE1d* overexpression on sumolylation under control and drought conditions, SUMO conjugates in both WT and ZmSCE1d-transgenic lines were detected by Western blotting using the anti-AtSUMO1 antibody. As shown in Figure 6, under control conditions, SUMO conjugates in both transgenic lines obviously increased (~50% increase averagely, relative to the WT) (Figure 6A,B). After 6 h-PEG treatment, accumulation of the SUMO conjugates in either WT or transgenic lines was significantly elevated (~180% increase for the transgenic lines averagely and ~90% increase for the WT) compared with their corresponding controls (Figure 6A,B). This suggested that *ZmSCE1d* overexpression facilitated sumoylation in transgenic *Arabidopsis* plants under drought stress.

### 2.7. Transcript Changes of Drought-Responsive Genes in ZmSCE1d-Trangenic Plants under Drought Stress

**Figure 7:**
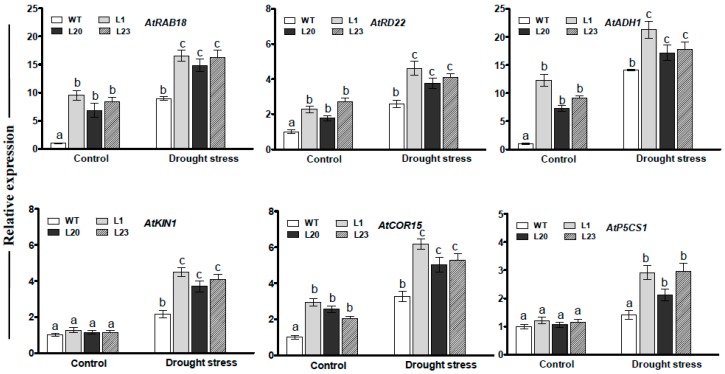
Transcriptional expression of drought-related genes in *ZmSCE1d*- overexpressing and wild type *Arabidopsis* plants under drought stress. Four-week-old plants from WT and three OE lines were treated with distilled water (control) or 20% PEG-6000 solutions, respectively, for 12 h, and leaves were sampled for RNA extraction, cDNA synthesis, and qPCR analysis. Data are presented as means ± SE and different letters on the histograms indicate significant differences (ANOVA; *p* < 0.05).

To understand effects of *ZmSCE1d* overexpression at transcription level, transcripts of several drought-related genes were examined by qPCR between WT and three transgenic lines under normal or drought conditions. As shown in Figure 7, under normal conditions, transcript levels of all the genes in these transgenic lines, except for *AtKIN1* and *AtP5CS1*, markedly increased compared to those in the WT plants (Figure 7). After 12 h of PEG treatment, the transcripts of all the genes were significantly elevated in these transgenic lines (increased by 1.5~5 folds, averagely) (Figure 7). These data implied that *ZmSCE1d* overexpression modulated expression of the drought-responsive genes during drought stress.

## 3. Discussion

Sumoylation regulates diverse biological processes, such as transcription, reproduction, and development, and environmental stress responses in plants [14,36]. However, the mechanisms underlying the sumoylation is involved in response to stress responses in maize are largely unknown. Our group has recently reported that the maize class-II SCE1 gene (*ZmSCE1e*) participated in drought and salinity stress responses [39]. In this study, we identified a maize class-I ZmSCE1 ortholog (*ZmSCE1d*), which was involved in drought response and tolerance possibly by modulating sumoylation levels, antioxidant capability, and drought-responsive gene expression.

There exists a single SCE gene (*AtSCE1*) in *Arabidopsis* and three SCE orthologs (*OsSCE1a*, *OsSCE1b*, and *OsSCE1c*) in rice [14]. In maize, SCEs contain seven genes (*ZmSCE1a*, *b*, *c*, *d*, *e*, *f*, *g*) that were likely produced by tandem duplication events [38]. However, the *ZmSCE1g* was identified as a pseudogene [38]. The established phylogenetic tree demonstrated that only four isotypes (*ZmSCE1a* to *ZmSCE1d*) of the seven maize SCE genes are the class-I members and other three members are clustered into the class-II and class-III, respectively, whereas all the three rice SCE1s belong to the clade-I (Figure 1). Functional characterization of the rice SCE1s showed that OsSCE1/OsSCE1a and OsSCE3/OsSCE1c had opposite effects on drought tolerance although they were clustered into the same class-I SCE1s [37]. Our previous study evidenced that the maize class-II SCE1e conferred drought and salt stress tolerance [39]. In the present study, we found that overexpression of the class-I member SCE1d conferred drought tolerance in transgenic *Arabidopsis* plants (Figure 3 and Figure 4), but had no significant effects on salinity stress tolerance. Interestingly, *Arabidopsis* AtSCE1 was shown to be required for viral infection in plants [41]. Together, these results imply that plant SCE1 isoforms have great functional divergences although they share similar structural features.

The ZmSCE1d-overexpressing *Arabidopsis* plants showed higher SOD and CAT activities and less MDA and H_2_O_2_ accumulations than the WT upon drought stress (Figure 5), implying that ZmSCE1d overexpression might reinforce antioxidant machinery for excess ROS scavenging and membrane lipid stability by activating antioxidant enzyme activities. Consistent with our findings, Karan and Subudhi (2012) reported that overexpression of the halophyte *Spartina alterniflora* SUMO E2 SCE gene (*SaSce9*) in *Arabidopsis* increased antioxidant enzymes SOD and CAT activities, and proline content, resulting in improving salinity and drought tolerance [42]. The improvement of antioxidant defense system is regarded as one of the general mechanisms of plant resistance to biotic and abiotic stressor and plant-eating predators [43,44,45]. For examples, alfalfa varieties with higher antioxidant enzyme activities are more tolerant to drought and salt stresses [46]. Also, Joo et al. (2019) evidenced that the OsSCE3-overexpressing rice plants with high antioxidant capability are positively associated with improved drought tolerance [37]. Together, ZmSCE1d-conferred drought tolerance of plants might be partially attributed to the activated antioxidant defense system under stress conditions. In further work, it is interesting to explore the involvement of *ZmSCE1d* in the antioxidant defense network via the multiple-omics approach using the *ZmSCE1d*-transgenic plants.

Overexpression of the *ZmSCE1d* increased levels of SUMO conjugates (Figure 6) and positively regulated drought tolerance of plants (Figure 4). Furthermore, *ZmSCE1d* overexpression activated transcripts of several drought-responsive genes upon drought stress (Figure 7). These data suggest that ZmSCE1d-mediated sumoylation is involved in cellular responses to environmental stresses. In agreement with this notion, it was reported that *AtSCE1* over-expression was positively correlated with sumoylation levels, which activated ABA- and stress-responsive gene expression in *Arabidopsis* [17]. Also, knockdown of the *AtSCE1* reduced sumoylation of cellular proteins and inhibited viral infection in *Arabidopsis* plants [41]. In addition, the SUMO conjugates rapidly increased when *Arabidopsis* plants were exposed to heat stress [18]. In tomato, the *SCE1*-silenced plants were more susceptible to a bacterial disease caused by *Clavibacter michiganensis* subsp. *michiganensis* [47]. These findings indicate that sumoylation plays a protective role in stress responses of plants. In consistent with the drought tolerance phenotypes, expression of several drought stress-related genes, including *AtRAB18*, *AtRD22*, *AtADH1*, *AtCOR15*, *AtKIN1*, and *AtP5CS1* was significantly upregulated under drought stress in the *ZmSCE1d* transgenic *Arabidopsis* lines (Figure 7). Noticeably, under normal conditions, transcript levels of the four genes *AtRAB18*, *AtRD22*, *AtADH1*, and *AtCOR15* in the transgenic lines were markedly increased (Figure 7). In particular, *AtRAB18* and *AtRD22* were evidenced to be regulated by transcription factors MYB and MYC [48]. Based on previous and our results, it is reasonable to speculate that under drought stress, ZmSCE1d promotes cellular sumoylation, which might affect activities of certain regulatory proteins such as MYC or MYB, and finally activated drought-responsive gene expression, resulting in improved drought tolerance in plants.

In summary, our data have demonstrated that *ZmSCE1d* overexpression improved drought tolerance likely by regulating sumoylation levels, antioxidant capability, and drought-responsive gene expression in transgenic plants. This study may facilitate our understanding of the mechanisms underlying the crop SCE-mediated sumoylation under drought stress. In future work, it will be needed to identify interacting partner(s) of the ZmSCE-mediated sumoylation, and thereby explore the exact molecular mechanisms in imparting drought tolerance in maize.

## 4. Materials and Methods

### 4.1. Plant Materials and Stress Treatment

The maize inbred line B73 and the *Arabidopsis* Columbia-0 (Col-0, as wild type) were used in this study. Plants were maintained in a growth room with 60–70% relative humidity, 25 ± 2 °C, and a photoperiod of 16 h light/8 h dark at a light intensity of around 200 µmol·m^−2^ s^−1^ [49]. For gene expression under drought treatment, two weeks old maize plants (with three true leaves) potted with soil contained a mixture of vermiculite and soddy soil (1:1, *v*/*v*) were treated by irrigating 200 mL distilled-water (Control), or 20% (*w*/*v*) PEG6000 (PEG) into soil in each pot (three plants for each pot). Subsequently, leaf samples were taken at 3, 6, 12, 24, and 48 h post treatment for expression analysis. For each treatment, nine plants were sampled. The uppermost leaf of each plant was collected and three leaves were mixed as a replicate. Three replicates were conducted in each treatment.

### 4.2. Sequence Analysis of ZmSCE1s

The open reading frame (ORF) of *ZmSCE1d* was amplified by the pair of primers ZmSCE1d-F and ZmSCE1d-R (Table S1), and the 483-bp PCR product was obtained and verified by sequencing. Sequence alignments between maize SCE1s and other species were done by DNASTAR. The phylogenetic tree among SCE1s from maize and other cereal species was constructed by MEGA 6 using standard parameters [50].

### 4.3. Quantitative Real-Time PCR

Isolation of total RNAs and synthesis of first-strand cDNAs were carried out as described by us [49]. Expression of *ZmSCE1a-ZmSCE1d* in maize was examined by quantitative real-time PCR (qPCR) using gene-specific primers (Table S1). The expression of the drought-responsive marker gene *DREB2A* in maize was also tested [40].

To assay the expression of several drought-responsive genes in transgenic *Arabidopsis* plants under normal and drought stress, qPCR analysis was also performed with the RNA samples isolated from four-week-old transgenic plants harvested after 12 h of 20% PEG-6000 treatment. Total RNA isolation and reverse transcription were performed as described above. PCR amplification was performed with primers specific for various stress-responsive genes (Table S1).

The qPCR assay was conducted in 96-well plates in triplicate on the StepOnePlus system (Applied Biosystems, Foster City, CA, USA) as described in our previous study [51]. Relative transcripts of each gene were calculated as reported previously [52]. The *ZmUbiquitin* was used as the reference gene for maize and *AtActin2* for *Arabidopsis*. In addition, three biological replicates were done in each qPCR experiment.

### 4.4. Vector Construction and Generation of Transgenic Plants

The ORF of *ZmSCE1d* was amplified using primers SCE1d-F2 and SCE1d-R2 and cloned into the pART7 vector [53]. Subsequently, the expression cassette harboring the 35S promoter and the *ZmSCE1d* coding region was sub-cloned into pART27 [53], resulting in the transformation construct pART27-35S-*ZmSCE1d*. The *Agrobacterium* GV3101 with the expression construct was transformed into *Arabidopsis* plants using the floral-dip method [54].

The transgenic *Arabidopsis* lines and their progenies were screened using kanamycin and confirmed by PCR as described previously [55]. To the end, six independent homozygous transgenic *Arabidopsis* lines (designated as L1, L4, L12, L16, L20, and L23) were developed.

### 4.5. Assessment of Osmotic and Drought Tolerance of the Transgenic Arabidopsis Lines

For osmotic stress tolerance analysis, surface-sterilized seeds of four transgenic *Arabidopsis* lines (L1, L4, L20, and L23) or wild type (WT) were germinated and cultured on 1/2 MS medium for 10 days, and then the seedlings were transferred to vertically grow on 1/2 MS medium supplemented with 0 or 300 mM mannitol in the growth chamber. The phenotypes of seedlings were recorded after 10 days.

For drought tolerance analysis, three-week-old WT and four transgenic *Arabidopsis* lines in soil were exposed to progressive drought by withholding water for 17 days. After that, fresh weight and remaining chlorophyll content in stressed WT and transgenic plants were determined as described previously [56]. These experiments were conducted in three biological replicates.

### 4.6. Determination of Total Chlorophyll, MDA and H_2_O_2_ Contents

Total chlorophyll content in leaves was determined by detecting absorbance at 663 and 645 nm in 80% acetone extracts as described in our previous study [56]. MDA content was measured using a MDA assay kit (BC0020, Solarbio, Beijing, China) according to the manufacturer’s instructions. Briefly, *Arabidopsis* leaves were ground with trichloroacetic acid (TCA) solution (5%, *w*/*v*) on ice, and reacted in thiobarbituric acid (TBA) solution (0.67%, *w*/*v*) for 30 min. After centrifuge (12,000× *g* for 15 min), absorbance of the resulting supernatant was measured at 532 and 600 nm, respectively. The MDA content was calculated as described previously [56].

H_2_O_2_ content was measured using a H_2_O_2_ assay kit (BC3595, Solarbio, Beijing, China) following the method of the manufacturer. In brief, leaf samples (0.1 g) were homogenized with 1 mL of 50 mM potassium phosphate buffer (pH 7.0). After centrifuge (4 °C, 8000× *g* for 10 min), the supernatant was reacted with NH_4_OH (15%, *v*/*v*) and TiCl_4_ (10%, *w*/*v*). The absorbance of the reaction mixture was measured at 415 nm. The H_2_O_2_ content was calculated following our previous method [49]. The experiments were performed three times, each repetition producing similar results.

### 4.7. Measurement of SOD and CAT Activities

The antioxidant enzymes SOD and CAT activities were measured using the detection kits (BC0170 and BC0200, respectively, Solarbio, Beijing, China). Briefly, *Arabidopsis* leaves were ground and homogenized in specific extraction buffers. After centrifuge, the resulting supernatants were used for enzyme activity assays. Total SOD and CAT activities were assayed by detecting changes in absorbance at 560 and 240 nm, respectively, following the kits’ protocols. The enzyme activities were expressed as U/g fresh weight, as described by us [39].

### 4.8. Sumoylation Analysis of Transgenic Arabidopsis Plants under Drought Stress

Four-week-old potted WT and OE transgenic *Arabidopsis* plants with uniform growth stage in the growth room under normal conditions were treated by irrigating 200 mL distilled-water (Control), or 20% (*w*/*v*) PEG6000 (PEG) into soil in each pot. After 6 h, total leaf proteins from control and stressed plants were extracted and sumoylation levels were examined by Western blotting assay as described previously [39]. The crude proteins were separated by SDS-PAGE (15%) and transferred onto PVDF membranes. The membranes were then blocked and blotted with a commercial anti-AtSUMO1 monoclonal antibody (Abcam, ab5316), and thereafter detected with a horseradish peroxidase (HRP)-conjugated IgG secondary antibody (CWBIO Co., Beijing, China). Finally, the HRP activity was detected using a 3,3′-diaminobenzidine (DAB) development kit (CWBIO Co., Beijing, China) and recorded by the Tanon 5200 Multi Chemiluminescent Imaging System (Shanghai, China). The experiments were carried out three times, each repetition producing similar results.

### 4.9. Statistical Analysis

The data were expressed as the means ± standard error (SE) and subjected to statistical analysis using the SPSS (version 17.0, SPSS Inc., Chicago, IL, USA). Data were analyzed by one-way analysis of variance (ANOVA), and means were compared by Duncan′s multiple range test at a significance level of *p* < 0.05.

## Supplementary Materials

The following are available online at https://www.mdpi.com/1422-0067/21/1/29/s1, Figure S1 Vector construction and expression levels of *ZmSCE1d* transgenes. A Expression cassette of the *ZmSCE1d*. B Transcription levels of *ZmSCE1d* in wild-type and *ZmSCE1*-overexpressing (OE) *Arabidopsis* lines by qPCR. The WT and six homozygous OE lines (named L1, L4, L12, L16, L20, and L23). Table S1 PCR primers used in this study.
