# Supplementary material for: The Maize Class-I SUMO Conjugating Enzyme ZmSCE1d Is Involved in Drought Stress Response
Source: Int J Mol Sci. 2019 Dec 19;21(1):29. doi: 10.3390/ijms21010029 (PMC6982253; doi:10.3390/ijms21010029)
Supplement: Supplementary file 1 [file ijms-21-00029-s001.pdf]

Table S1 PCR primers used in this study.

Primer name	Primer sequence*	Use
ZmSCE1a-QF<br>ZmSCE1a-QR	5'-CCGCACGGTTTCGTCGCA-3'<br>5'-CGAAAGACAGACTGTTCCAGAAGGATAG-3'	qPCR for *ZmSCE1a* expression
ZmSCE1b-QF<br>ZmSCE1b-QR	5'-GGCAAGCAAGGGACTGAC-3'<br>5'-GCTGGTCTCCAACCACTG-3'	qPCR for *ZmSCE1b* expression
ZmSCE1c-QF<br>ZmSCE1c-QR	5'-TGTTAAGCAGATTCTAGTTGGAATACAA-3'<br>5'-GAACACGTCGCTTATATTCTGTT-3'	qPCR for *ZmSCE1c* expression
ZmSCE1d-QF<br>ZmSCE1d-QR	5'-CAGGAACACGTCGCTTATATTCTGC-3'<br>5'-TCTGCCTCTCAATTCTGAAC-3'	qPCR for *ZmSCE1d* expression
AtRD22-QF<br>AtRD22-QR	5'-ATGGCGATTCGTCTTCCTCTGATC-3'<br>5'-ACTCCGCCTTTACCTACTTGGACG-3'	qPCR for *AtRD22* expression
AtKIN1-QF<br>AtKIN1-QR	5'-ATGTCAGAGACCAACAAGAATGCC-3'<br>5'-CTACTTGTTCAGGCCGGTCTTG-3'	qPCR for *AtKIN1* expression
AtRAB18-QF<br>AtRAB18-QR	5'-ATGGCGTCTTACCAGAACCGTCCA-3'<br>5'-ACCACCACTTTCCTTGTGGAGTTG-3'	qPCR for *AtRAB18* expression
AtADH1-QF<br>AtADH1-QR	5'-ATGTCTACCACCGGACAGATT-3'<br>5'-CGAGTGGCAATGACGACACTC-3'	qPCR for *AtADH1* expression
AtCOR15-QF<br>AtCOR15-QR	5'-CTTCTTTCCACAGCGGAGCCA-3'<br>5'-GATGTTGCCGTCACCTTTAGCG-3'	qPCR for *AtCOR15* expression
AtP5CS1-QF<br>AtP5CS1-QR	5'-TCGTGGTCCAGTCGGGGTCG-3'<br>5'-TGGGAATGTCCTGATGGGTGTAA-3'	qPCR for *AtP5CS1* expression
ZmSCE1d-F2<br>ZmSCE1d-R2	5′-AGGAATTCATGTCTGGAGGGATCGCG-3′<br>5′-ACAAGCTTTCAGACCAGAGCAGGATATTG-3′	Construction of *ZmSCE1d* over-expression vector
ZmDREB2A-QF<br>ZmDREB2A-QR	TGCAGCCCGGAAGGAAG<br>TTGAGTATTGTCAGGCCCCC	qPCR for *ZmDREB2A* expression
AtActin2-F<br>AtActin2-R	5'-TTGTGCTGGATT CTGGTGATGG-3'<br>5'-CCGCTCTGCTGTTGTGGTG-3'	qPCR for *AtActin2* expression

*The underlined nucleotides constitute *Eco*RI (GAATTC) and *Hin*dIII (AAGCTT) restriction enzyme digestion sites.
